# Root Endophytic Fungal Community and Carbon and Nitrogen Stable Isotope Patterns Differ among *Bletilla* Species (Orchidaceae)

**DOI:** 10.3390/jof7020069

**Published:** 2021-01-20

**Authors:** Xinhua Zeng, Ziyi Ni, Haixin Diao, Kai Jiang, Chao Hu, Li Shao, Weichang Huang

**Affiliations:** 1 Shanghai Chenshan Plant Science Research Center, Chinese Academy of Sciences, Chenshan Botanical Garden, Shanghai 201620, China; zengxinhua@csnbgsh.cn (X.Z.); niziyi@csnbgsh.cn (Z.N.); dhxtx1216@163.com (H.D.); Jiangkai@csnbgsh.cn (K.J.); huchao@csnbgsh.cn (C.H.); shaoli@csnbgsh.cn (L.S.); 2 Shanghai Key Laboratory of Plant Functional Genomics and Resources, Shanghai Chenshan Botanical Garden, Shanghai 201602, China; 3 College of Landscape Architecture, Fujian Agriculture and Forestry University, Fuzhou 350002, China

**Keywords:** *Bletilla*, stable isotope, fungal diversity, symbiotic relationship, mycorrhizal colonization, ITS amplicon sequencing

## Abstract

Orchids of the genus *Bletilla* are well-known ornamental plants and sources of traditional medicine in Asia that rely on the symbiotic relationship with root endophytic fungi throughout their whole life cycle. However, little is known about their fungal partners, infection pattern, and pathways of carbon gain. We investigated carbon and nitrogen stable isotope patterns in different organs of three *Bletilla* species, identified the root endophytic fungal community composition, and determined mycorrhizal colonization rates. The three *Bletilla* species were comprised by a polyphyletic group which belongs to different trophic modes, such as saprotroph, pathotroph, and symbiotroph; however, the dominant species and their abundances varied among *Bletilla* spp. Mycorrhizal infection rates also varied among *Bletilla* species, with *B. striata* (65% ± 25%) being significantly higher than those of *B. formosana* (35% ± 16%) and *B. ochracea* (22% ± 13%). Compared with surrounding autotrophic plants, all *Bletilla* spp. were significantly enriched in ^13^C with *B. striata* to a significantly higher level than other two *Bletilla* species. Among different organs, stems had higher δ^13^C values, while leaves and flowers had higher δ^15^N and total N content values across all three species. Our results indicate that the symbiotic relationship of *Bletilla* and its root endophytic fungi is not strictly specific. Although mycorrhizal infection rates were highly variable, the three *Bletilla* species had the same infection pattern with hyphae penetrating the cortex cell by the pathway cell. Different *Bletilla* species have different strategies for C allocation among plant organs. These findings provide new insights into the ecological adaptation of orchids and will contribute to *Bletilla* germplasm conservation and sustainable utilization.

## 1. Introduction

Mycorrhizae are an ancient, widespread association between fungi and land plants [1]. As a consequence of their tiny dust-like seeds having only marginal amounts of carbon reserves within the embryos, orchids rely on mycorrhizal fungi throughout their life cycle, but especially during the seed germination and seedling recruitment periods [2,3,4]. Most orchids have thick fleshy roots with few lateral roots and root hairs, which are not conducive to the absorption of immobile mineral nutrients such as P and Fe [5,6]. However, the external hyphae of orchid mycorrhizae can offset these deficiencies in absorbing ability [7]. Researchers have found that root endophytic fungi can not only transport carbohydrates and break down cellulose in the matrix, but can also directly provide nutrients and hormones (e.g., amino acids, gibberellins and jasmonate) for plant growth [5,8,9]. In addition, root endophytic fungi were found to promote the absorption ability of macroelements and microelements by orchids [10,11,12]. Meanwhile, root endophytic fungi can also enhance orchid plants to produce metabolites such as antibiotics, phenolic compounds, peroxidase, and hydrolase, which enhance the disease resistance and stress tolerance of orchid plants [13,14].

The trophic relationship between orchids and root endophytic fungi is an important issue of mutualistic interactions. In the early stage, orchid seed germination and seedling establishment under natural conditions are totally dependent on carbon and nutrients supplied by fungal partners [15], and this mode of nutrition is referred to as initial mycoheterotrophy [16]. However, as mature plants, based on their utilization of carbon sources, orchids are divided into three types: (1) photosynthetic, in which orchid plants are chlorophyllous and obtain carbon fully through photosynthesis [1,12]; (2) partially mycoheterotrophic, in which orchids are also chlorophyllous but obtain C both from their own photosynthesis and their fungal symbionts [10,15]; (3) mycoheterotrophic, in which orchids are achlorophyllous and thus entirely dependent on fungal C throughout their life cycles. Among the approximately 28,000 orchid species, there are more than 235 fully mycoheterotrophic species [16]. Researchers have found that partial mycoheterotrophy is not a strictly static nutritional pattern but an evolutionary metastable trait, in which the two carbon sources coordinate with each other, enabling better adaption to changing environments [7,17]. Liebel et al. [12] found that the C and N exchange between *Goodyera repens* and its mycorrhizal fungus was greatly affected by light availability and chlorophyll concentrations. Preiss et al. [18] found that *Cephalanthera damasonium* and *Cephalanthera rubra* had different mycoheterotrophic levels under different light conditions, with a higher fungal C proportion at low irradiance and higher photosynthetic C proportion at high irradiance. Bellino et al. [19] found that *Limodorum abortivum* derived carbon mainly from fungi in its natural condition; however, photosynthesis played a supplementary role when the fungal carbon source was limited.

Stable isotope natural abundance analysis, together with the molecular identification of mycorrhizal partners, is a powerful approach to assessing the nutritional mode of mature orchids [10,20]. By using carbon and nitrogen stable isotopes, it is possible to evaluate nutrient fluxes in ecosystem processes under field conditions and trace the source of specific nutrients naturally occurring, by utilizing isotopic differences between plant- and fungus-derived C and N [21,22]. Due to the discrimination and fixation of ^12^C vs. ^13^C by photosynthesis, autotrophic plants are depleted for ^13^C compared with neighbouring mycoheterotrophs [23,24]. Meanwhile, different functional groups of fungi forming orchid mycorrhizae can obtain different soil-derived and/or plant-derived C and nutrients, which thus form different stable isotope abundance patterns in fungal tissues [23,25] and consequently give associated orchids isotopic signatures that resemble their fungal associates [26]. Researchers have found that orchids associated with ectomycorrhizal fungi (such as achlorophyllous orchids) [27,28] and saprotrophic wood-decomposers or litter-decaying fungi [29,30] were enriched in ^13^C and ^15^N isotopes in comparison to neighbouring autotrophic plants and that mature partially mycoheterotrophic orchids (i.e., green forest orchids associated with ectomycorrhizal fungi) were positioned between fully mycoheterotrophic orchids and autotrophic plants [10]. Compared with mycoheterotrophic orchids, stable isotope natural abundance for photosynthetic orchids varied among species [22]. *Listera ovata* and *Orchis purpurea* were found to exhibit ^13^C enrichment [10,31], while significant ^13^C depletion was found in the orchid tribes Orchideae and Cranichideae [32,33]. However, Gebauer and Meyer [10] found that some rhizoctonia-associated orchids exhibit ^13^C abundance equivalent to that of autotrophic reference plants.

*Bletilla* Rchb.f is a genus of terrestrial orchids distributed across northern Burma, China, and Japan, and it belongs to the tribe Epidendreae in the subfamily Orchidoideae within Orchidaceae [34]. There are six species around the world, with four species distributed within China. Owing to their high ornamental and medicinal value, *Bletilla* species are widely used as ornamental plants and as traditional medicinal materials in Asia [35]. For this reason, these plants have frequently been over-collected in the past and are now considered as threatened. All *Bletilla* species in China are now listed by the Convention on International Trade in Endangered Species of Wild Fauna and Flora [36,37]. In order to protect *Bletilla* germplasm resources and enable sustainable utilization of plant resources, it is necessary to conduct protective cultivation of *Bletilla* and understand the trophic relationships of *Bletilla* with their mycorrhizal partners. At present, research on *Bletilla* species has mainly focused on germplasm resources, genetic diversity, evaluation of its active ingredients, artificial propagation, and thus, little is known about their mycorrhizal partners and nutritional mode. Although few studies have investigated the mycorrhizal associations of *Bletilla*, they utilized the traditional morphological identification methods according to the conidiogenous structure or sexual propagation characteristics of fungi, yielding the identification of very few microorganisms and failed to reflect the reality of mycorrhizal associations.

In this study, we investigated the carbon and nitrogen stable isotope natural abundance of three sympatric mature greenish *Bletilla* species (*Bletilla striata* (Thunb. ex A. Murray) Rchb. f., *Bletilla ochracea* Schltr. and *Bletilla formosana* (Hayata) Schltr.) by using stable isotope analysis, and we also analysed the composition and specificity of mycorrhizal associations using molecular identification methods. Our main objectives are to answer the following questions. (1) What are the mycorrhizal partners of *Bletilla*? (2) Do the community structure, colonization rate, and infection pattern of root endophytic fungi vary among the three *Bletilla* species? (3) Are there any differences among *Bletilla* species or different organs within a plant concerning the stable isotope characteristics of carbon and nitrogen?

## 2. Materials and Methods

### 2.1. Study Site and Plant Species

Samples were collected from the *Bletilla* germplasm resource nursery located in Shanghai Chenshan Botanical Garden (31°04′ N and 121°11′ E) at 71.4 m elevation. The area has a subtropical monsoon climate with a mean annual precipitation of 1213 mm and a mean annual temperature of 15.6 °C. The soil matrix was composed of mountain clay, river sand, and vermiculite with a ratio of 1.5:1:1 and a pH of 6.5–7.5 (in the top 0–5 cm). The cultivated plants of *B. striata*, *B. ochracea*, *B. formosana* were taken from wild of Chongyi County (Jiangxi), Debao County (Guangxi) and Tainan County (Taiwan) three years ago, respectively, and were already fully adapted to the site environment.

### 2.2. Sampling

Sampling was conducted during the 2018 flowering season in the germplasm resource nursery of Shanghai Chenshan Botanical garden. Three 1 m × 1 m plots were randomly established in the *B. striata*, *B. ochracea*, and *B. formosana* sites. In each plot, two roots per plant from five orchid plants were collected. Collected roots from each plot were mixed and kept cold during transport to the laboratory for further analysis. The roots were divided into three parts, one used for mycelium colonization observation, one for endophytic fungal molecular identification, and another for C and N stable isotope abundance analysis. For each species, we sampled fresh top leaves, stems and flowers of three flowering individuals during its flourishing florescence. Meanwhile, leaves of five autotrophic reference plants under the same microclimate were also sampled on 14 May, 2018. The autotrophic reference species were chosen based on the criteria described by Gebauer and Meyer [10]. The five reference species are *Erigeron annuus* (L.) Pers., *Bischofia javanica* Bl., *Morus alba* L., *Broussonetia papyrifera* (Linn.) LHer. ex Vent. and *Conyza canadensis* (L.) Cronq. In total, 60 samples from three *Bletilla* species (stem, leaf, flower and root samples from each species) and 25 leaf samples from five autotrophic reference species were collected in this study.

### 2.3. Observation of Mycelium Colonization

The mycorrhizal status was assessed by viewing fine washed root cross-sections under a stereoscopic microscope. Mycorrhizal roots were fixed in a 50% ethanol:formaldehyde:acetic acid solution (90:5:5) for microscopy observation. Root pieces were dehydrated in a graded ethanol series (75%, 85%, 95%, and 100% ethanol), embedded in paraffin, cut transversely and sliced into 8 μm thick sections that were stained with safranin-O/fast green. The sections were dehydrated using an alcohol-xylene series and mounted with neutral balsam, and fungal colonization was observed under a light microscope. Sections were attributed to four colonization categories as described in Gonneau et al. [11]: C0, no pelotons in the section’s cells; C1, pelotons in 1–30% of the cells; C2 pelotons in 31–60% of the cells; C3, pelotons in >60% of the cells. Mean colonization was estimated by averaging the percentage colonization of all investigated sections according to the following equation:

(1) Colonization rate (%)=(15%×NC1+15%×NC2+15%×NC3)NT

where *N_C1_*, *N_C2_*, *N_C3_* and *N_T_* represent the amount of C1, C2, C3 and all sections.

### 2.4. Molecular Identification of Root Endophytic Fungi

Roots were rinsed with tap water, sonicated to remove adhering soil and dirt, and sterilized as follows: roots were rinsed with sterile water for 30 s and 70% ethyl alcohol for 2 min, soaked in 2.5% sodium hypochlorite for 5 min, transferred to 70% ethyl alcohol for 30 s, and finally washed with sterile water three times. The roots were cut into small cross-sections with sterile scissors, and ten to twelve sections per sample were selected for genomic DNA extraction and purification using the FastDNA Spin Kit for Soil (MP Biomedicals, Irvine, CA, USA) according to the manufacturer’s protocol. The nuclear ribosomal internal transcribed spacer (ITS) region was amplified with the fungal-specific primers ITS1F and ITS4 [12]. The PCR amplification was performed as follows: initial denaturation at 95 ℃ for 3 min, followed by 35 cycles of denaturing at 95 °C for 30 s, annealing at 55 °C for 30 s and extension at 72 °C for 45 s, and single extension at 72 °C for 10 min, and end at 10 °C The PCR reactions were performed in triplicate 20 μL mixture containing 2 μL of 10 ×Pyrobest Buffer, 2 μL of 2.5 mM deoxyribonucleotide triphosphates (dNTPs), 0.8 μL of each primer (5 μM), 0.2 μL of Pyrobest DNA polymerase (TaKaRa), 10 ng of template DNA, and finally ddH_2_O up to 20 μL. The PCR product was extracted from 2% agarose gel and purified using the AxyPrep DNA Gel Extraction Kit (Axygen Biosciences, Union City, CA, USA) according to manufacturer’s instructions and quantified using Quantus™ Fluorometer (Promega, Madison, WI, USA).

All positive PCR products were purified with the AxyPrep DNA Gel Extraction Kit (Axygen Biosciences, Union City, CA, USA) and sequenced paired-ends on an Illumina MiSeq platform (Illumina, San Diego, CA, USA) according to the standard protocols from Majorbio Bio-Pharm Technology Co. Ltd. (Shanghai, China). The generated paired-end reads were merged once, but because the reads were longer than 300 bp, the paired-end reads could not be merged without overlap. Thus, we used single-end long reads for further analysis. The raw reads were deposited into the NCBI Sequence Read Archive (SRA) database (Accession Number: SRP218217). Raw fastq files were demultiplexed and quality-filtered with Trimmomatic (version 0.19.6, https://github.com/OpenGene/fastp) according to the following criteria: 300 bp reads that were truncated at any site receiving an average quality score of <20 over a 50 bp sliding window, and the truncated reads shorter than 50 bp were discarded, while reads containing ambiguous characters were also discarded. Operational taxonomic units (OTUs) were clustered with a 97% similarity cut-off using UPARSE (version 7.1, http://drive5.com/uparse/) and chimeric sequences were removed using USEARCH (version 7.0, http://www.drive5.com/usearch/). The taxonomy of each ITS rRNA gene sequence was analysed with the RDP Classifier algorithm (http://rdp.cme.msu.edu/) against the UNITE version 8.0 ITS rRNA database (https://unite.ut.ee/) using a confidence threshold of 70%. Fungal OTUs were classified into different putative trophic strategies following the classification of the FunGuild v1.0 (http://www.stbates.org/guilds/app.php).

### 2.5. Analysis of Stable Isotope Abundance and N Concentration

Stem, leaf, flower, and root samples were washed with deionized water, oven-dried at 105 °C, ground into a fine power and stored in a desiccator fitted with silica gel until subsequent analysis. Relative C and N isotope abundances and N content were measured using an elemental analyser (vario PYRO cube; Elementar Analysensysteme GmbH, Langenselbold, Germany) coupled with a continuous flow isotope ratio mass spectrometer (IsoPrime100, Elementar UK Ltd., Stockport, UK), as described in Bidartondo et al. [38]. Relative isotope abundances are denoted as δ values, which were calculated according to the following equation:

(2) δ13C or δ15N=(RsaRst−1)×1000‰ 

where *R_sa_* and *R_st_* are the ratios of heavy isotopes to light isotopes in the samples and the respective standards. Standard gases were calibrated with USGS40 and USGS41a for carbon and nitrogen isotopes, provide by the United States Geological Survey (USGS). The reproducibility and accuracy of the isotope abundance measurements were routinely controlled by measures of laboratory standard acetanilide. The calculation of C and N concentrations in the samples followed the protocol of Gebauer and Schulze [39]. For relative C and N isotope natural abundance analyses, acetanilide was routinely analysed with variable sample weights once every 12 samples.

Enrichment factors (ε_s_) for all samples were calculated according to the following equation:

(3) $$\varepsilon_{s}=\delta_{s}-\delta_{\mathrm{ref}}$$

where δ_S_ is the relative isotope abundance of a sample of *Bletilla*, and δ_ref_ is the mean isotope abundance of all autotrophic reference plants.

### 2.6. Statistical Analyses

All statistical analyses were performed using SPSS 16.0 for Windows (SPSS Inc., Chicago, IL, USA). Before analysis, all variables were checked for normality by Shapiro–Wilks tests and for homogeneity of variance by Levene’s tests. One-way ANOVA with Tukey HSD post hoc comparisons or Tamhane’s T2 test were used if data were normally distributed. In other cases, Kruskal–Wallis nonparametric tests with Mann–Whitney U tests for post hoc comparisons were used. We checked the differences in δ^13^C and δ^15^N values among the autotrophic reference plants and *Bletilla* species as well as differences in δ^13^C and δ^15^N values and N content among different organs of each *Bletilla* species. Significant difference in three alpha diversity indexes and coverage among different *Bletilla* species were also compared in this study. Significance was defined at the 95% confidence level.

## 3. Results

### 3.1. Mycelium Colonization of Three Bletilla Species

**Figure 1:**
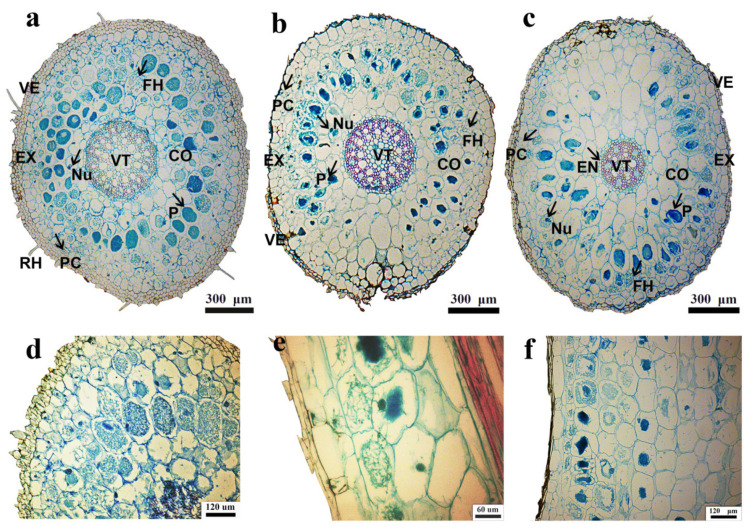
Root cross-sections and infection position of mycorrhizal fungi of *B. striata* (**a**,**d**), *B. ochracea* (**b**,**e**), and *B. formosana* (**c**,**f**). CO, cortex; EN, endodermis; EX, exodermis; FH, hyphae; P, phloem; PC: pathway cell; Nu: nucleus; VE: velamen; VT: vascular tissue; RH: root hair. The arrow points to pathway cell.

The cortical parenchyma of *B. striata*, *B. ochracea*, and *B. formosana* were found to be colonized by typical fungal pelotons at the flowering stage. Mycorrhizal infection rates varied among *Bletilla* species, with *B. striata* (65% ± 25%) (Figure 1a) having significantly higher infection rates than those of *B. formosana* (35% ± 16%) (Figure 1b) and *B. ochracea* (22 ± 13%) (Figure 1c). However, the three *Bletilla* species had the same infection pattern of mycorrhizal fungi, in which hyphae penetrated the pathway cell, and later the cortex, and finally colonized in the cortex cell (Figure 1d–f). Meanwhile, all three *Bletilla* species had two mycelial morphologies of clumpy pelotons and filamentous pelotons.

### 3.2. Fungal Community Found in Bletilla Roots

**Figure 2:**
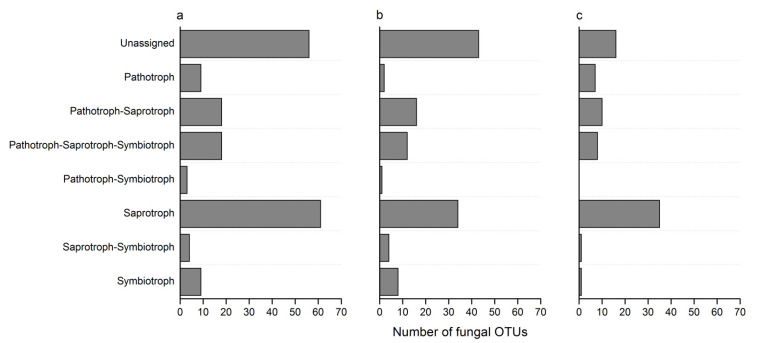
Frequency distribution displaying the number of operational taxonomic units (OTUs) belonging to the different trophic guilds identified in the roots of *B. striata* (**a**), *B. ochracea* (**b**), and *B. formosana* (**c**).

Illumina MiSeq sequencing yielded a total of 380,832 sequences with a mean length of 265 bp that passed the quality filtering and could be assigned to the nine samples. The number of sequences per individual orchid varied from 31,677 to 60,571. A total of 347 operational taxonomic units (OTUs) were identified using a 3% dissimilarity cutoff and removed chimeric sequences as well as global singletons. After discarding non-fungal sequences and data flattened, 273 OTUs could be assigned to root-associated fungal endophytes, which belonged to 50 orders in eight phyla. The orders with the highest number of OTUs were Hypocreales (27 OTUs), unclassified_c__Agaricomycetes (25 OTUs), Pleosporales (24 OTUs), Helotiales (24 OTUs) and Eurotiales (22 OTUs). Using FunGuild database, there were 122 OTUs, 77 OTUs, and 62 OTUs that could be assigned to the putative life strategies for *B. striata, B. ochracea*, and *B. formosana*, respectively (Figure 2). For *B. striata*, the putative life strategies with the highest number of OTUs were saprotroph (61 OTUs), pathotroph-saprotroph (18 OTUs), pathotroph-saprotroph-symbiotroph (18 OTUs), symbiotroph (9 OTUs) and pathotrophs (9 OTUs) (Figure 2a). For *B. ochracea*, the putative life strategies with the highest number of OTUs were saprotroph (34 OTUs), pathotroph-saprotroph (16 OTUs), pathotroph-saprotroph-symbiotroph (12 OTUs) and symbiotroph (8 OTUs) (Figure 2b). For *B. formosana*, the putative life strategies with the highest number of OTUs were saprotroph (35 OTUs), pathotroph-saprotroph (10 OTUs) and pathotroph-saprotroph-symbiotroph (8 OTUs) (Figure 2c). Among these OTUs, about 51 OTUs could be considered as putative species of OMF: there were related to Aspergillaceae (*Aspergillus* and *Penicillium*), Tulasnellaceae (*Tulasnella*), Ceratobasidiaceae (*Rhizoctonia*), Cortinariaceae (*Gymnopilus*), Cucurbitariaceae (*Pyrenochaeta*), Glomerellaceae (*Colletotrichum*), Helotiaceae (*Meliniomyces*), Herpotrichiellaceae (Exophiala), Hypocreaceae (*Trichoderma*), Myxotrichaceae (*Oidiodendron*), Nectriaceae (*Neocosmospora*, *Fusarium*, *Cylindrocarpon*), Pleosporaceae (*Alternaria*), Saccharomycetales_fam_Incertae_sedis (*Candida*) and Serendipitaceae (*Serendipita*).

### 3.3. Diversity of Root Endophytic Fungi for Three Bletilla Species

Fungal community richness (Sobs) and alpha diversity (Shannon and Simpson) index values were compared for different *Bletilla* species at the OTU level (Table 1). The sobs estimator indicated that fungal community richness of *B. striata* was significantly higher than that of *B. formosana* (*p* < 0.05). The Shannon indices showed that *B. striata* had the highest while *B. ochracea* had the lowest root endophytic fungal diversity, and there did not exist significant differences among the three species (*p* > 0.05). Meanwhile, Simpson’s indices also exhibited no significant differences among the three species. The coverage scores were highly comparable for all species, ranging from 99.97% to 99.99%, indicating that the sequencing depth was adequate to reliably describe the mycorrhizal fungi associated with the three species, and no significant differences were observed among the three species.

### 3.4. Community Composition of Root Endophytic Fungi among Bletilla Species

**Figure 3:**
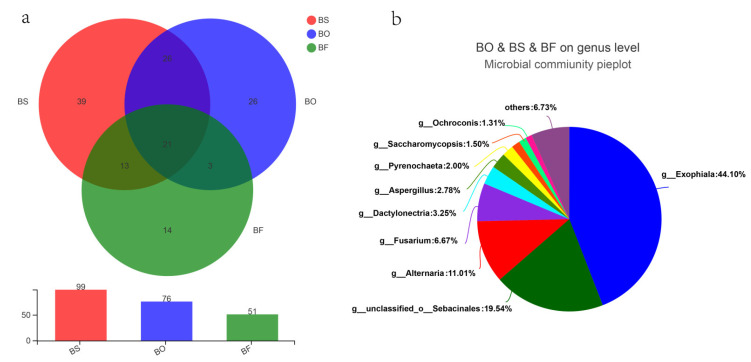
The genus-level composition of root endophytic fungi for three *Bletilla* species (**a**) and the shared genus among three *Bletilla* species (**b**). BS, BO, and BF represent *B. striata*, *B. ochracea*, and *B. formosana*, respectively.

**Figure 4:**
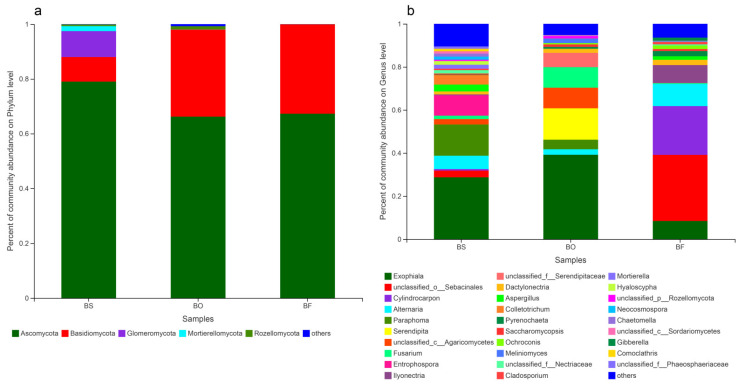
Relative abundances of different fungal phyla (**a**) and genera (**b**) in root endophytic fungi among three *Bletilla* species. BS, BO, and BF represent *B. striata*, *B. ochracea*, and *B. formosana*, respectively.

A total of 99, 76, and 51 fungal genera were identified for *B. striata*, *B. ochracea* and *B. formosana*, respectively, and 21 of them were common to all three species. There are 39, 26, and 14 fungal genera specific to *B. striata*, *B. ochracea*, and *B. formosana*, respectively (Figure 3a). The shared 21 genus among three *Bletilla* species belong to *Exophiala* (44.1%), unclassified_o_Sebacinales (19.54%), *Alternaria* (11.01%), *Fusarium* (6.67%), *Dactylonectria* (3.25%), *Aspergillus* (2.78%), *Pyrenochaeta* (2.00%), *Ochroconis* (1.31%) and others (6.73%) (Figure 3b). At the phyla level, *B. striata* was dominated by Ascomycota (79.02%), Basidiomycota (8.98%) and Glomeromycota (9.46%), while *B. ochracea* and *B. formosana* were dominated by Ascomycota (66.21% and 67.28%, respectively) and Basidiomycota (31.83% and 32.62%, respectively) (Figure 4a). The predominant genera for *B. striata* were *Exophiala* (28.70%), *Paraphoma* (14.52%), *Entrophospora* (9.46%), *Alternaria* (6.19%), *Colletotrichum* (4.47%) and *Aspergilus* (3.17%). For *B. ochracea*, the predominant genera were *Exophiala* (39.17%), *Serendipita* (14.58%), unclassified_c_Agaricomycetes (9.57%), *Fusarium* (9.55%), unclassified_f_Serendipitaceae (6.65%), *Paraphoma* (4.46%), and *Alternaria* (2.55%). For *B. formosana*, the predominant genera were unclassified_o_Sebacinales (30.69%), *Cylindrocarpon* (22.64%), *Alternaria* (10.31%), *Exophiala* (8.44%), *llyonectria* (8.30%), and *Dactylonectria* (2.43%) (Figure 4b).

**Figure 5:**
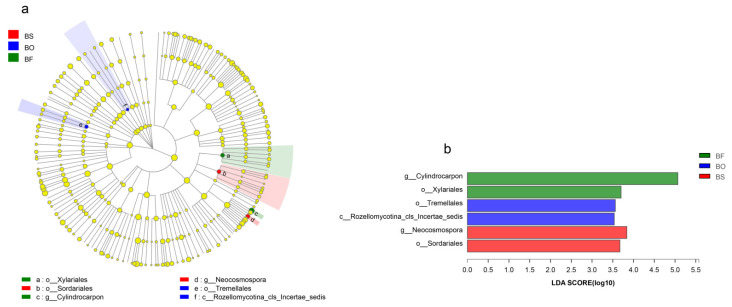
Cladogram showing the phylogenetic distribution of mycorrhizal fungi associated with different species of *Bletilla* (**a**). Indicator fungi with LDA scores of 2 or greater in mycorrhizal fungi associated with different *Bletilla* species (**b**). Differently coloured regions represent different *Bletilla* species (green, *B. formosana*; blue, *B. ochracea*; red, *B. striata*). Circles indicate phylogenetic levels from phyla to genus. The diameter of each circle is proportional to the abundance of the group. BS, *B. striata*; BO, *B. ochracea*; BF, *B. formosana*.

In addition to α-diversity analysis, we used LEfSe discriminant analysis to identify specialized communities in samples. Groups are shown in cladograms, and LDA scores of 2 or greater were performed by LEfSe (Figure 5). For *B. striata*, two groups of fungi were significantly enriched, namely Sordariales (order) and *Neocosmospora* (genus). For *B. ochracea*, two fungi were significantly enriched, namely Tremellales (order) and Rozellomycotina_cls_Incertae_sedis (class). For *B. formosana*, two fungi groups were also significantly enriched, namely *Cylindrocarpon* (genus) and Xylariales (order).

### 3.5. Stable Isotope Natural Abundance of Different Organs for Three Bletilla Species

**Figure 6:**
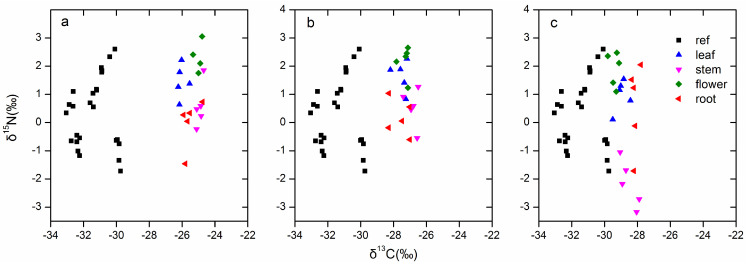
Overview of δ^13^C and δ^15^N values of stem, leaf, flower, and root tissues from *Bletilla*, and leaf tissue from autotrophic reference plants (ref) in this study. (**a**) represents *B. striata*; (**b**) represents *B. ochracea*; (**c**) represents *B. formosana*.

The overall results of the C and N stable isotope abundance analysis for different organs of *Bletilla* and autotrophic reference plants (ref) are shown in Figure 6. Significant differences (*p* < 0.05, Tamhane’s T2 test) were detected in δ^13^C among autotrophic references and *Bletilla*. For all three *Bletilla* species examined, the mean δ^13^C values of all organs were significantly higher than those of autotrophic reference plants (*p* < 0.05, Table 2). The δ^13^C values of different organs for *B. striata*, *B. ochracea*, and *B. formosana* ranged from −26.23‰ to −24.69‰, −28.3‰ to −26.5‰ and −29.81‰ to −27.8‰, with mean values of −25.37‰ ± 0.12‰, −27.32‰ ± 0.11‰ and −28.76‰ ± 0.13‰, respectively (Figure 6). Significant differences existed in the δ^13^C values among the three *Bletilla* species (*p* < 0.05, Table 2). For *B. striata* and *B. ochracea*, stem and flower tissues had significantly higher δ^13^C values than leaf and root tissues, while in *B. formosana*, roots had significantly higher δ^13^C values than leaves and flowers did (*p* < 0.05, Table 3).

For δ^15^N, *B. striata* and *B. ochracea* had higher mean values than the autotrophic reference plants and *B. formosana* did. However, there was no significant difference among each other (Table 2). For different organs, the δ^15^N values for *B. striata*, *B. ochracea* and *B. formosana* ranged from 3.06‰ to −1.46‰, 2.66‰ to −0.6‰ and 2.48‰ to −3.17‰, with the flower and leaf tissues relatively higher than stem and root tissues (Table 3).

### 3.6. Enrichment Factors and N Concentrations among Different Species and Different Organs of Bletilla

**Figure 7:**
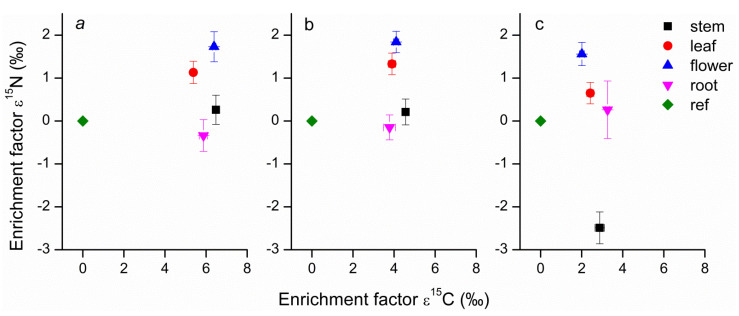
Mean enrichment factors (ε) for ^13^C and ^15^N of stem, leaf, flower, and root tissues of three *Bletilla* species, and leaf tissue of autotrophic reference plants (ref). For the reference plants, mean ε is zero by definition. (**a**) represents *B. striata*; (**b**) represents *B. ochracea*; (**c**) represents *B. formosana*. Error bars represent standard errors of the mean enrichment factors for ^13^C or ^15^N (n = 5).

Relative enrichment factors (ε) for ^13^C and ^15^N were calculated for different organs of *Bletilla* species. For all three *Bletilla* species examined, the ε^13^C values of different organs were all higher than those of the autotrophic reference plants. Meanwhile, the mean ε^13^C values of the three *Bletilla* species were highest for *B. striata* (6.03‰), followed by *B. ochracea* (4.09‰) and then *B. formosana* (2.64‰), and there were significant differences among these three. For all *Bletilla* species, the ε^15^N values varied considerably among the different organs, with higher values for flower and leaf tissues than for stem and root tissues (Figure 7).

**Figure 8:**
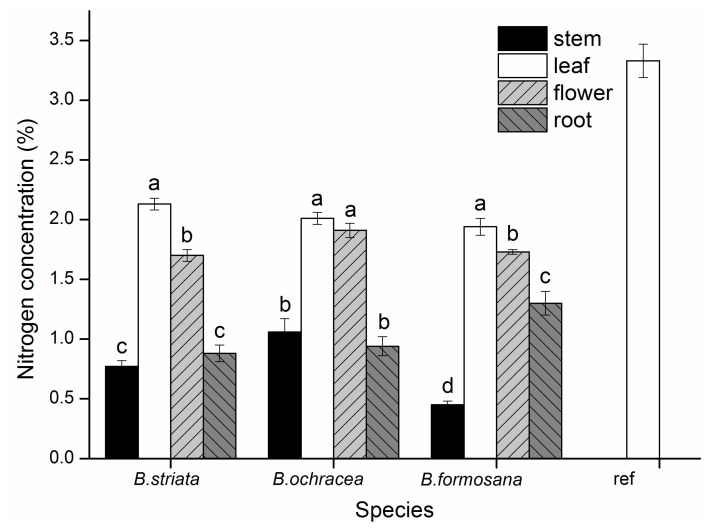
Nitrogen concentration of different organs for three *Bletilla* species and autotrophic references plants (ref). Differences among organs for each *Bletilla* species were compared by multiple comparison analysis. Error bars represent standard errors of the mean nitrogen concentrations (n = 5).

Nitrogen concentrations of the different organs of *Bletilla* and leaf of autotrophic reference plants were compared in this study. The leaf of autotrophic reference plants had a higher nitrogen concentration compared with different organs of *Bletilla*. Nitrogen concentrations among different organs varied considerably for each *Bletilla* species. For all three *Bletilla* species, leaf and flower tissues had significantly higher nitrogen concentrations than root and stem tissues did (*p* < 0.05, Figure 8).

## 4. Discussion

### 4.1. Composition and Diversity of Root Endophytic Fungi

Our molecular analysis of root endophytic fungi revealed that all three sympatric *Bletilla* species were mainly associated with fungi from Ascomycota and Basidiomycota, with their abundances varying among *Bletilla* species. For *B. striata*, 79.02% of mycorrhizal fungi were Ascomycota, while 8.98% were Basidiomycota. For *B. ochracea* and *B. formosana*, the percentage ratios were not very different; 66.21% and 67.28% were assigned to Ascomycota, while 31.83% and 32.62% belonged to Basidiomycota. Based on ITS rRNA sequences, 21 fungal genera were shared by all the three species, 42 genera shared by two species and 79 genera associated with a single species. Meanwhile, the composition of root endophytic fungi differed significantly among the three species. We observed a predominance of *Exophiala* (28.70%) and *Paraphoma* (14.52%) in association with *B. striata*, a predominance of *Exophiala* (39.17%) and *Serendipita* (14.58%) for *B. ochracea*, and a predominance of unclassified_o_Sebacinales (30.69%), *Cylindrocarpon* (22.64%) and *Alternaria* (10.31%) for *B. formosana*. The three sympatric *Bletilla* species shared some fungi species, with the dominant fungal species and their abundances varying among the three congeners; this may reflect distinct mycorrhizal preferences for specific host species, or alternatively, this pattern could result from the host undergoing natural selection for specific mycorrhizal associations [40]. After transplantation from the field, the orchids may have lost their original symbionts through the replacement of old roots, while new roots would then be colonized by local fungal species from the nursery. Once an appropriate fungal partner is found, the plants can then fine-tune their physiology to adapt to that particular fungus [40]. Another possible explanation is that the three *Bletilla* species were transplanted from different localities with different mycobiont compositions. If different localities harbour different fungal species that can form mutualisms with *Bletilla* species, then this would influence differences in mycobiont compositions among species [41].

Examples of the high specificity of mutualistic interactions between orchids and root endophytic fungi are widespread in nature [42]. *Corallorhiza maculata* and *C*. *mertensiana* are found to form mycorrhizas exclusively with fungi in the Russulaceae [43], *Hexalectris spicata* specifically forms ectomycorrhizal associations with fungi in the Sebacinaceae [25], and *Epipactis helleborine* forms associations almost exclusively with the genus *Wilcoxina* [44]. However, in this study, the relationship of root endophytic fungi to *Bletilla* species appeared unlikely to be particularly specific. This might be a consequence of *Bletilla* species occurring among highly diverse fungal communities and having the ability to form mycorrhizal mutualisms with different partners, which provides opportunities for tolerating new environmental conditions and changes in resource availability. Consider *B. ochracea* for example; Tao et al. [45] found that *Epulorhiza*, *Ceratorhiza*, *Sebacina*, and *Phomopsis* were the dominant genera in Guizhou, while Liu et al. [46] found *Epulorhiza* and *Sebacina* were the most common fungi. However, in our study, *Exophiala* and unclassified *Tremellomycetes* were the predominant genera, while *Epulorhiza* and *Sebacina* were very rare in mycorrhizal roots. Another possible reason for this is that fungal endophytes in root cortical tissues of *Bletilla* comprise a polyphyletic group which contains saprophytic fungi, pathogenic fungi, and symbiotic fungi, and only a few fungi can form mycorrhizal with *Bletilla*. This finding demonstrates that the specificity of interactions between *Bletilla* and mycorrhizal symbionts is not particularly strict.

### 4.2. Isotope Signature and Nutrient Gain from Fungal Partners

Our data show that highly significant differences existed among the C and N isotope signatures of all groups tested (i.e., reference autotrophic plants and three *Bletilla* species). With respect to δ^13^C values, *Bletilla* species were significantly higher than autotrophic reference plants (with 6.03‰, 4.08‰ and 2.64‰ enrichment for *B. striata*, *B. ochracea* and *B. formosana*, respectively). Meanwhile, *B. striata* had significantly higher mean δ^13^C values than did *B. ochracea* and *B. formosana*, indicating *B. striata* derived more C resources from fungi compared to the other two species. With respect to δ^15^N, *B. striata* and *B. ochracea* were significantly enriched compared with autotrophic plants and *B. formosana*. This indicates that there was interspecific variation in nutrient physiology among *Bletilla* species. The variation in ^13^C enrichment among different *Bletilla* species might be attributable to their different fungal partner compositions, fungal colonization rates or photosynthesis rates. In this study, the dominant fungal species and their abundances varied among the three *Bletilla* species. Meanwhile, photosynthesis rates also varied among the three *Bletilla* species, with *B. formosana* (9.03 ± 0.28 μmol m^−2^ s^−1^) having a higher rate than that of *B. striata* (8.42 ± 0.1 μmol m^−2^ s^−1^) and *B. ochracea* (5.61 ± 0.16 μmol m^−2^ s^−1^). Chen et al. found that partial mycoheterotrophy is not a strictly static nutritional pattern but an evolutionary metastable trait, in which photosynthetic C and fungal C coordinate with each other [7].

Among different organs, stems had relatively higher ^13^C enrichment compared with leaves and flowers. As the carbon capture and metabolism of plants still require further research, we can only speculate on the mechanisms behind the differences in C enrichment among plant organs. Stems of three *Bletilla* species may be produced using resources accumulated the previous year or derived from associated fungi. Stems were formed in early spring, at which time C was probably derived or at least in part from plant reserves, in agreement with the findings of Vallius [47] and Cernusak et al. [48]. Vallius [46] found that non-evergreen orchids such as *Dactylorhiza* species used nutrients stored in their tubers to form stems, flowers, and fruits. Cernusak et al. [48] found that plant reserves were usually enriched in ^13^C, which may contribute to the high δ^13^C values observed at the early stages of young stem and leaf formation. Gonneau et al. [11] also found that photosynthetic C plays little part in mixotrophic plants’ below-ground organs and reserves, which had high δ^13^C values, in sharp contrast with autotrophic plants. Considering *B. striata* and *B. ochracea*, flowers had significantly higher δ^13^C values than leaves did, which might indicate that more fungal C or less photosynthetic C was allocated into flowers compared with leaves. However, the situation is different for *B. formosana*, which has lower δ^13^C values of flowers than that of leaves. This indicates that different *Bletilla* species have different strategies for C allocation among plant organs over their flowering periods, as reported by Johansson et al. [49]. Different patterns of C assimilation, reallocation or recycling of previously assimilated compounds can cause intra-plant variation in δ^13^C [11]. Leaf and flower tissues had significantly higher δ^15^N and total N content values than stem tissues did for the three *Bletilla* species. Therefore, we can deduce that there was more nitrogen transferred to flower and leaf tissues compared with other organs.

### 4.3. The Functional Roles of Root-Associated Fungal Endophytes

Symbiosis mycorrhizae are a widespread association between fungi and orchid, and play a vital role throughout the whole life cycle of orchid [1]. Root endophytic fungi can not only provide nutrients and hormones for plant growth, but also enhance plant disease resistance and stress tolerance [9,13]. Using the FunGuild database, there were 122, 77, and 62 OTUs could be assigned to the putative life strategies for *B. striata*, *B. ochracea* and *B. formosana*, respectively. For all three *Bletilla* species, the putative life strategies with the top three were saprotrophs, pathotroph-saprotroph, and pathotroph-saprotroph-symbiotroph. The proportions of saprotrophic fungi for three *Bletilla* species ranged from 44% to 56%, which were similar to the results of Cevallos et al. [50]. Cevallos et al. found that saprotrophs occupied the largest proportion of 45% in the roots of neotropical epiphytic orchids in the southern Ecuadorian Andes. Functional types of fungi are closely related with trophic modes of orchids. Selosse and Roy [51] found that autotrophic orchids generally associated with a variety of saprotrophic *Rhizoctonia* spp., while mycoheterotrophic orchids tended to be highly specialized to a narrow range of ectomycorrhizal fungi. Mycorrhizal symbionts of partially mycoheterotrophic orchids are in a transitional stage [10].

Researchers found that fungal communities potentially contributed to orchid adaptation to changing environmental conditions [52], so it is necessary to evaluate the ecological roles of root-associated fungal endophytes and their effect on orchids. However, the ecological roles of many root-associated fungal endophytes are largely unknown and diverse. For examble, *Fusarium* spp. is assigned as pathotroph-saprotroph-symbiotroph based on FunGuild. *Fusarium* spp. has been reported to cause root rot in commercial orchid species such as vanilla [53], and it is also described as mycorrhizal fungi in *Bletilla striata* [54]. Similarly, some saprotrophic fungi that we detected in this study, such as *Aspergillus*, *Penicillium*, *Gymnopilus*, *Pyrenochaeta*, *Trichoderma* and *Neocosmospora*, are found to form orchid mycorrhizal [55,56]. Meanwhile, some pathotrophic fungi such as Ilyonectria, *Cylindrocarpon*, *Alternaria* and *Cylindrocarpon* are highly pathogenic in ginseng and other plants, but there did not find infecting orchid root [57].

At present, the functional role of endophytic fungi in orchids remains poorly characterized, and there were 56, 43, and 16 OTUs unassigned for *B. striata*, *B. ochracea*, and *B. formosana*, accounting for 45.9%, 35.85%, and 20.51%, respectively, for each species. There are three possible reasons for this. First, due to the limited sequencing technology, the taxonomic classification of OTUs may not reach the species level in a public database [52,58]. Second, the putative life strategy was assigned only to OTUs with taxonomic assignment at ‘species’ level based on the currently published literature or authoritative website data which do not contain all fungal species. Third, distinguishing between mycorrhizal and non-mycorrhizal fungi in orchids is challenging, because the functional roles of endophytic fungi are continuously being discovered [54,59]. Thus, it is necessary to reinforce the fungal taxonomy and improve the DNA databases, which will help us better understand the characterization of fungal communities associated with orchids [60]. In addition, further studies of electron microscopy and isotope technique to assess the infection morphology or their interaction with orchids are also needed.

## 5. Conclusions

Orchids of the genus *Bletilla* are of great economic value in Asia as ornamental plants and traditional medicine. Identifying the symbiotic relationship of *Bletilla* with its root endophytic partners is critical for plant growth and horticultural application. We investigated the mycorrhizal colonization of three adult greenish *Bletilla* species (*B. striata*, *B. ochracea*, *B. formosana*), and analyzed the composition and specificity of root endophytic fungal partners. Additionally, we compared the carbon and nitrogen stable isotope natural abundance of different species and different organs within a plant. Mycorrhizal infection rates were highly variable among *Bletilla* species; however, the three *Bletilla* species had the same infection pattern of mycorrhizal fungi, with hyphae penetrating the cortex cell by the pathway cell. Mature *Bletilla* plants are partially mycoheterotrophic, with the level of mycoheterotrophy varying among species within the genus. *Bletilla* species also have different strategies for C allocation among plant organs, with stems mainly using resources accumulated in the previous year or derived from associated fungi, while leaves are mainly derived from photosynthates. Additionally, the symbiotic relationship between *Bletilla* and its root endophytic partners is not strictly specific. Our results have important implications for our understanding of the fungus–host relationship and providing useful information in *Bletilla* germplasm conservation and sustainable utilization.

## Figures and Tables

**Table 1:** Alpha diversity estimates of root endophytic fungi associated with *Bletilla*. Data were analyzed by one-way ANOVAs with Tukey HSD post hoc comparisons. Significant differences (*p* < 0.05) among different species for each index are indicated by differences in lowercase letters.

	Sobs	Shannon	Simpson	Coverage (%)
*B. striata*	94 ± 15 ^a^	2.35 ± 0.48 ^a^	0.26 ± 0.12 ^a^	99.97 ^a^
*B. ochracea*	73 ±9 ^ab^	1.76 ± 0.31 ^a^	0.32 ± 0.06 ^a^	99.98 ^a^
*B. formosana*	39 ± 5 ^b^	1.89 ± 0.45 ^a^	0.30 ± 0.14 ^a^	99.99 ^a^

Same lowercase letters within a column indicate no significant difference between *Bletilla* species.

**Table 2:** Comparison of mean δ^13^C and δ^15^N values among the autotrophic reference plants and three *Bletilla* species (*p* < 0.05).

	Autotrophic References	*B. striata*	*B. ochracea*	*B. formosana*
δ^13^C	−31.40 ± 0.22 ^d^	−25.37 ± 1.02 ^a^	−27.32 ± 1.14 ^b^	−28.76 ± 0.33 ^c^
δ^15^N	0.33 ± 0.25 ^a^	1.02 ± 0.24 ^a^	1.14 ± 0.22 ^a^	0.33 ± 0.39 ^a^

Different lowercase letters within a row indicate significant differences in δ^13^C or δ^15^N values among autotrophic references and three *Bletilla* species (*p* < 0.05).

**Table 3:** Comparison of δ^13^C and δ^15^N values (mean±SE) among different organs for each *Bletilla* species.

	δ^13^C				δ^15^N			
	Stem	Leaf	Flower	Root	Stem	Leaf	Flower	Root
*B. striata*	−24.92 ± 0.08 ^a^	−26.02 ± 0.12 ^c^	−25.01 ± 0.09 ^a^	−25.53 ± 0.20 ^b^	0.59 ± 0.35 ^bc^	1.46 ± 0.26 ^ab^	2.06 ± 0.35 ^a^	−0.01 ± 0.37 ^c^
*B. ochracea*	−26.84 ± 0.16 ^a^	−27.5 ± 0.18 ^b^	−27.3 ± 0.13 ^a^	−27.62 ± 0.29 ^b^	0.54 ± 0.3 ^b^	1.66 ± 0.25 ^a^	2.17 ± 0.25 ^a^	0.18 ± 0.28^b^
*B. formosana*	−28.52 ± 0.23 ^ab^	−28.98 ± 0.17 ^bc^	−29.39 ± 0.12 ^c^	−28.14 ± 0.09 ^a^	−2.16 ± 0.37 ^b^	0.98 ± 0.25 ^a^	1.89 ± 0.27 ^a^	0.59 ± 0.68 ^a^

The same lowercase letters within a row indicates there was no significant difference in δ^13^C or δ^15^N values among different organs for each *Bletilla* species. Significance was defined at the 95% confidence level.

## Data Availability

All sequence data are available in NCBI GenBank following the accession numbers in the manuscript.
